# Gene expression signatures of human senescent corneal and conjunctival epithelial cells

**DOI:** 10.18632/aging.205113

**Published:** 2023-09-28

**Authors:** Koji Kitazawa, Akifumi Matsumoto, Kohsaku Numa, Yasufumi Tomioka, Zhixin A. Zhang, Yohei Yamashita, Chie Sotozono, Pierre-Yves Desprez, Judith Campisi

**Affiliations:** 1 Buck Institute for Research on Aging, Novato, CA 94945, USA; 2 Department of Ophthalmology, Kyoto Prefectural University of Medicine, Kyoto 6020841, Japan; 3 Lawrence Berkeley National Laboratory, Berkeley, CA 94720, USA

**Keywords:** cellular senescence, cornea, conjunctiva, Stevens-Johnson syndrome, limbal stem cell deficiency

## Abstract

Purpose: This study aimed to investigate the senescent phenotypes of human corneal and conjunctival epithelial cells.

Methods: We examined cell morphology, senescence-associated β-galactosidase (SA-β-gal) activity, cell proliferation, and expression of senescence markers (p16 and p21). RNA sequencing analysis was conducted to compare gene expression profiles between senescent and non-senescent cells. Finally, the potential involvement of senescent cells in the pathogenesis of ocular surface diseases was investigated.

Results: X-irradiated corneal and conjunctival epithelial cells exhibited typical senescence phenotypes, i.e., flattened morphologies, increased SA-β-gal activity, decreased cell proliferation, and increased expression of senescence markers, p16 and p21. RNA-seq analysis revealed substantial differences in gene expression profiles between senescent corneal (SCo) and conjunctival epithelial cells (SCj). Moreover, SCj were detected in pathological conjunctival tissues associated with limbal stem cell deficiency (LSCD) due to Stevens-Johnson syndrome or chemical burns, potentially being involved in abnormal differentiation.

Conclusion: This study highlights the cellular and molecular characteristics of senescent ocular surface cells, particularly in SCj that show abnormal keratin expression, and their potential roles in severe ocular surface diseases and pathology.

## INTRODUCTION

Aging is a universal process, and, with the global aging of the society, attention has been focused on diseases that occur during aging. Understanding the various biological changes associated with aging is very important for developing new treatments for age-related diseases [1]. Many hallmarks have been proposed for the biological age-related changes, including genomic instability, telomere attrition, epigenetic alterations, loss of proteostasis, disabled macroautophagy, deregulated nutrient-sensing, mitochondrial dysfunction, cellular senescence, stem cell exhaustion, altered intercellular communication, chronic inflammation, and dysbiosis [2].

Senescent cells accumulate during aging and contribute to pathological conditions in various ways [3, 4]. Recent research on cellular senescence has reported that senescent cells acquire a proinflammatory phenotype, termed the senescent-associated secretory phenotype (SASP) [5], which can alter the surrounding microenvironment over time [6, 7]. Therefore, effort has been made to understand the phenotype of senescent cells, and several culture models of cellular senescence have been generated.

The concept of cellular senescence appeared from the studies of replicative senescence, which causes shortened telomeres upon propagation of cells in culture [8]. It was also reported that senescent cells can be induced through oncogene overexpression [9], DNA damage response induced upon X-irradiation [10] or by the chemotherapeutic drug doxorubicin [11]. Determining the role of senescent ocular surface cells could increase our knowledge of the pathology of refractory age-related ocular surface diseases, including dry eye and limbal stem cell deficiency (LSCD). Here, we induced cellular senescence in human corneal and conjunctival epithelium using X-irradiation, and analyzed gene expression profiles of each cell type to determine the characteristics of senescent ocular surface cells.

## RESULTS

### Characteristics of X-irradiated corneal and conjunctival epithelial cells

**Figure 1:**
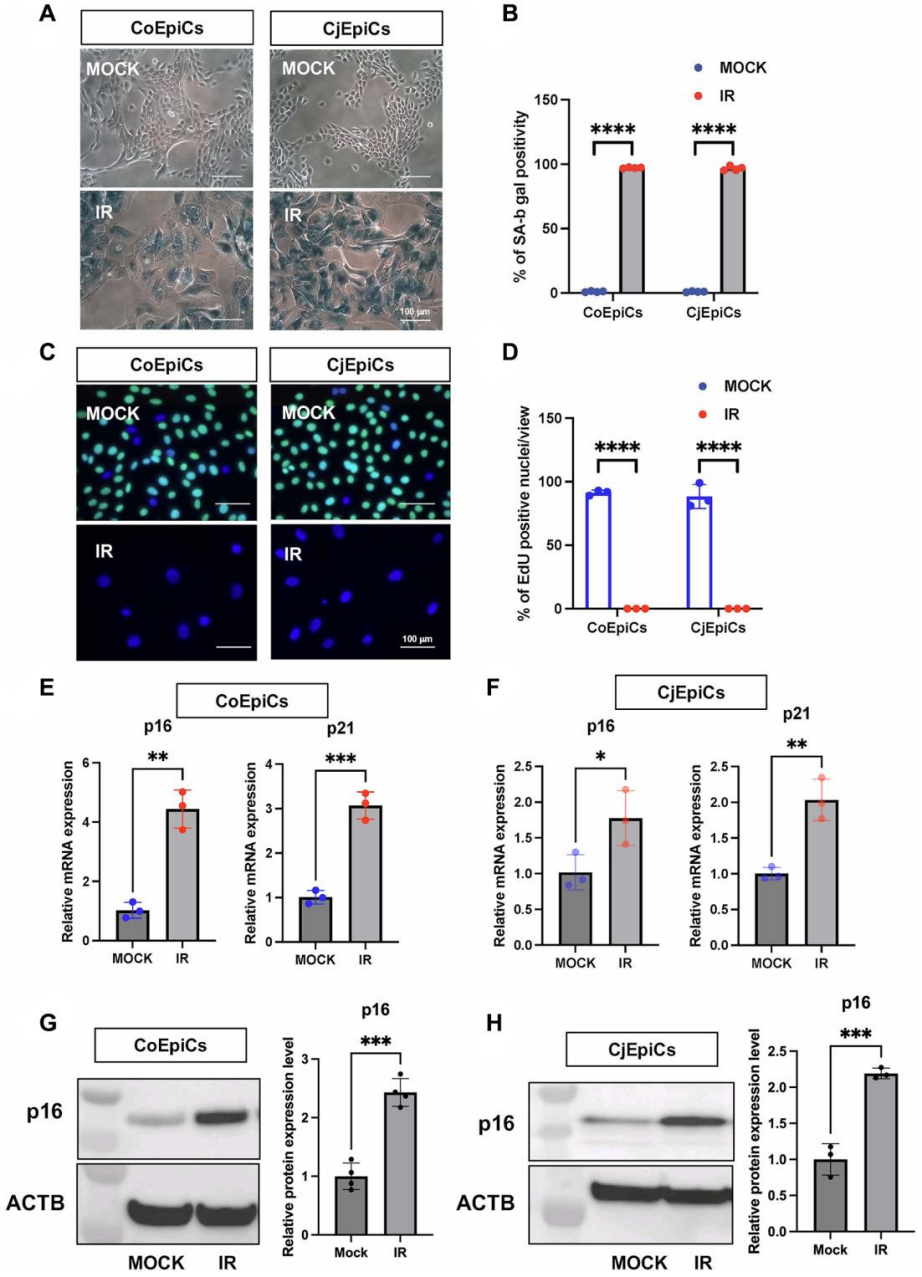
**Characteristic of X-irradiated corneal and conjunctival epithelial cells.** (**A**) SA-β-gal expression in irradiated (IR) or non-irradiated (MOCK) CoEpiCs and CjEpiCs. (**B**) Percentage of SA-β-gal positivity (*n* = 4). (**C**) EdU incorporation in irradiated or non-irradiated CoEpiCs and CjEpiCs. (**D**) Percentage of EdU positivity (*n* = 3). (**E**) RNA expression analysis of *p16* and *p21* in irradiated or non-irradiated CoEpiCs (*n* = 3). (**F**) RNA expression analysis of *p16* and *p21* in irradiated or non-irradiated CjEpiCs (*n* = 3). (**G**) Protein expression analysis of p16 in irradiated or non-irradiated CoEpiCs (*n* = 3). (**H**) Protein expression analysis of p16 in irradiated or non-irradiated CjEpiCs (*n* = 3). Gene expression was normalized to the housekeeping gene *GAPDH*. ^*^p < 0.05; ^**^p < 0.01; ^***^p < 0.001. Scale bars indicate 100 μm.

We first determined the characteristics of X-irradiated (IR) corneal epithelial cells (CoEpiCs) and conjunctival epithelial cells (CjEpiCs). As controls (mock), non-irradiated CoEpiCs and CjEpiCs showed homogenous populations of small cells. However, X-irradiated CoEpiCs and CjEpiCs appeared more elongated and flattened, and significantly bigger than non-irradiated cells (Figure 1A). SA-β-gal in irradiated CoEpiCs and CjEpiCs was revealed in blue (Figure 1A), and the positivity of SA-β-gal staining was 97.1% and 96.6%, respectively, compared to 1.1% and 1.0% in non-irradiated cells (Figure 1B). EdU-labeling was not detectable in irradiated CoEpiCs and CjEpiCs, whereas 91.3% and 88.3 % of non-irradiated CoEpiCs and CjEpiCs were EdU-positive (Figure 1C, 1D). We next examined the expression of the senescence markers *p16* and *p21*. Using qRT-PCR, both *p16* and *p21* were significantly elevated in irradiated CoEpiCs and CjEpiCs compared to non-irradiated cells (Figure 1E, 1F), and western blotting for p16 expression showed similar differences between cell populations (Figure 1G, 1H), suggesting that irradiated CoEpiCs (SCo) and CjEpiCs (SCj) are indeed senescent.

### Investigation of senescent corneal and conjunctival epithelial cells using RNA-Seq

**Figure 2:**
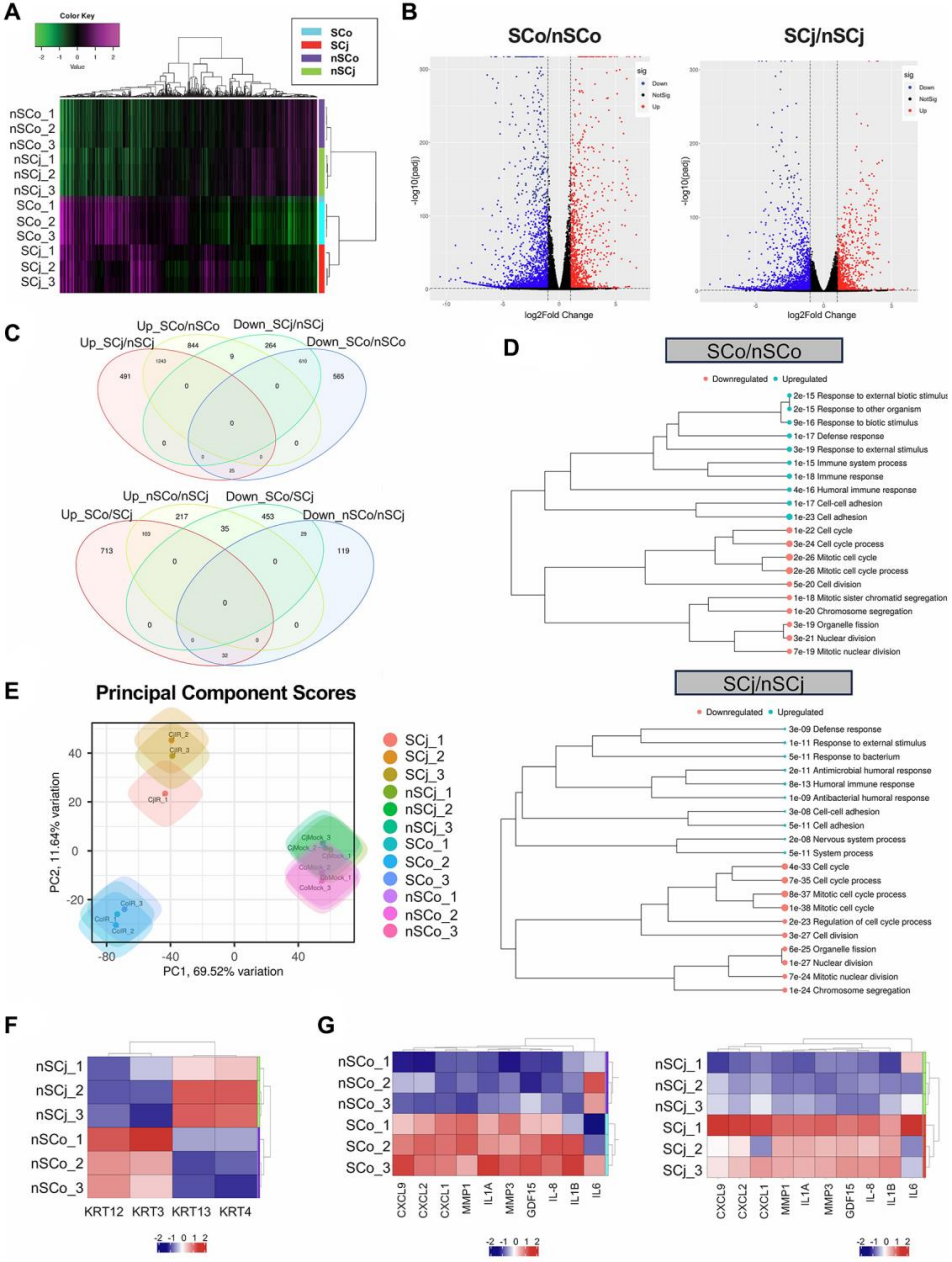
**Comprehensive transcriptional analysis of senescent corneal and conjunctival epithelial cells.** (**A**) Clustering analysis of senescent CoEpiCs (SCo), non-senescent CoEpiCs (nSCo), senescent CjEpiCs (SCj) and non-senescent CjEpiCs (nSCj) (*n* = 3 for each condition). (**B**) Volcano plot comparing SCo and nSCo (left panel), as well as SCj and nSCj (right panel). (**C**) Analysis of differentially expressed genes in SCo, nSCo, SCj, and nSCj. (**D**) Gene ontology analysis of upregulated or downregulated genes comparing SCo and nSCo (top panel), as well as SCj and nSCj (bottom panel). (**E**) Principal component analysis of SCo, nSCo, SCj, and nSCj. (**F**) Expression analysis of cell-specific keratin genes. (**G**) Expression of senescence-associated secretory phenotype (SASP)-related genes comparing SCo and nSCo (left panel), as well as SCj and nSCj (right panel).

We next used these SCo and SCj to extract RNA in order to investigate by RNA-Seq the full spectrum of genes regulated upon senescence induction compared to control non-senescent CoEpiCs (nSCo) and CjEpiCs (nSCj). The total number of reads were 71,168,793 and 64,004,656 for SCo and nSCo, and 56,316,010 and 64,181,750 for SCj and nSCj. In order to validate the data collected, clustering analysis was performed for SCo, nSCo, SCj, and nSCj, and we detected a high level of clustering between nSCo and nSCj, and between SCo and SCj (Figure 2A). Our RNA sequencing analysis revealed significant differences in gene expression between SCo/SCj vs. nSCo/nSCj (Figure 2B). A total of 3,295 genes were found to be differentially expressed (DEGs), with 1,200 genes upregulated and 2,095 genes downregulated in SCo compared to nSCo (fold change >2 and adjusted *p*-value < 0.05) (Figure 2C). A total of 2,642 genes were found to be differentially expressed, with 883 genes upregulated and 1,759 genes downregulated in SCj compared to nSCj (fold change >2 and adjusted *p*-value < 0.05) (Figure 2C). Among the upregulated genes, expression changes for both *p16* and *p21* were confirmed. For SCo/nSCo, the log2FC values were 1.00 and 1.05 for *p16* and *p21*, respectively. For SCj/nSCj, the log2FC values were 0.17 and 0.58 for *p16* and *p21*, respectively.

We then conducted gene ontology analysis and identified several biological processes that were significantly enriched in the DEGs. Pathway analysis showed that the upregulated genes were enriched in pathways related to cellular metabolism, cell signaling, and immune response, while the downregulated genes were enriched in pathways related to DNA repair, cell cycle regulation, and chromatin organization, in both SCo and SCj (Figure 2D). Principal component analysis (PCA) was also performed to identify patterns of gene expression variability across samples (Figure 2E). nSCo showed high expression of corneal epithelium-specific keratins, keratin 3 and keratin 12, whereas nSCj exhibited high expression of conjunctival epithelium-specific keratins, keratin 4 and keratin 13 (Figure 2F). There were therefore cell-specific differences in the expression of these keratins. Moreover, SCo and SCj displayed significantly different gene expression profiles compared to nSCo and nSCj (Figure 2E). Both, SCo and SCj showed high expression of inflammatory cytokines, chemokines and matrix metalloproteinases (Figure 2G), suggesting that senescent ocular surface cells acquire a senescence-associated secretory phenotype (SASP).

### Comparison of gene expression patterns between senescent corneal and conjunctival epithelial cells

**Figure 3:**
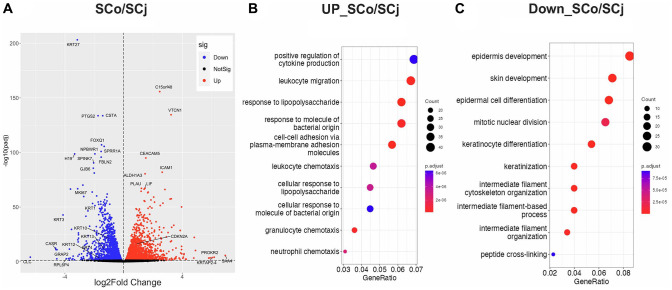
**Comparison of gene expression patterns between senescent corneal and conjunctival epithelial cells.** (**A**) Volcano plot comparing SCo and SCj. (**B**) Gene ontology analysis of upregulated genes in SCo compared to SCj. (**C**) Gene ontology analysis of downregulated genes in SCo compared to SCj. Abbreviations: SCo: senescent corneal epithelial cells; nSCo: non-senescent corneal epithelial cells; SCj: senescent conjunctival epithelial cells; nSCj: non-senescent conjunctival epithelial cells.

In order to further investigate the differences in their biological functions, we compared the transcriptomic profiles of both senescent cell populations, corneal epithelial cells and conjunctival epithelial cells. Our results showed that these two types of senescent cells have distinct gene expression patterns (Figure 3A). Indeed, to further decipher the biological processes that were affected by cellular senescence in these two cell types, we performed pathway analysis. Interestingly, we found that the inflammatory pathway was more enriched in SCo (Figure 3B), while epidermis development, keratinocyte differentiation, and keratinization pathways were more enriched in SCj (Figure 3C). Interestingly, TGF-β signaling appears to play a more significant role in the senescent phenotype of the corneal cell type compared to the conjunctival cell type (Supplementary Figure 1). These findings suggest that cellular senescence differentially affect the physiological functions of corneal and conjunctival epithelial cells, which could have important implications for the pathology of ocular surface diseases.

### Pathological involvement of senescent conjunctival epithelial cells

**Figure 4:**
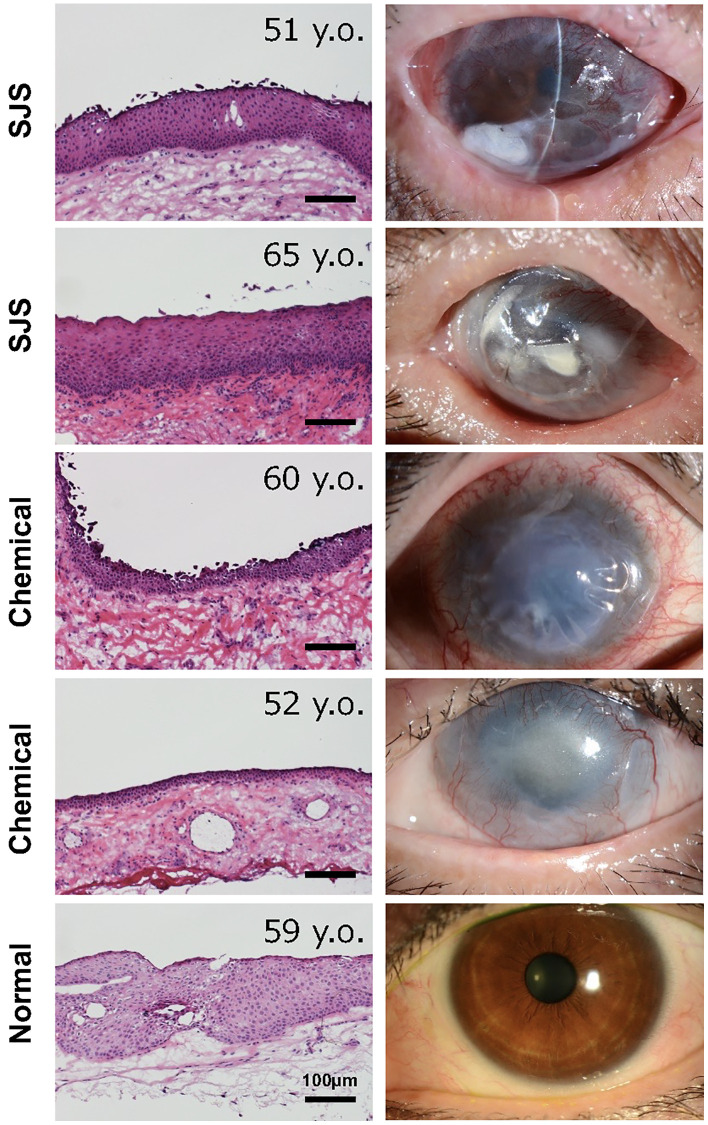
**Histology of conjunctival tissues from patients with limbal stem cell deficiency.** Representative ocular images of two cases with Stevens-Johnson syndrome (SJS), two cases with chemical injury, and one healthy subject. The histological sections were stained with hematoxylin and eosin stain. Scale bars indicate 100 μm.

**Figure 5:**
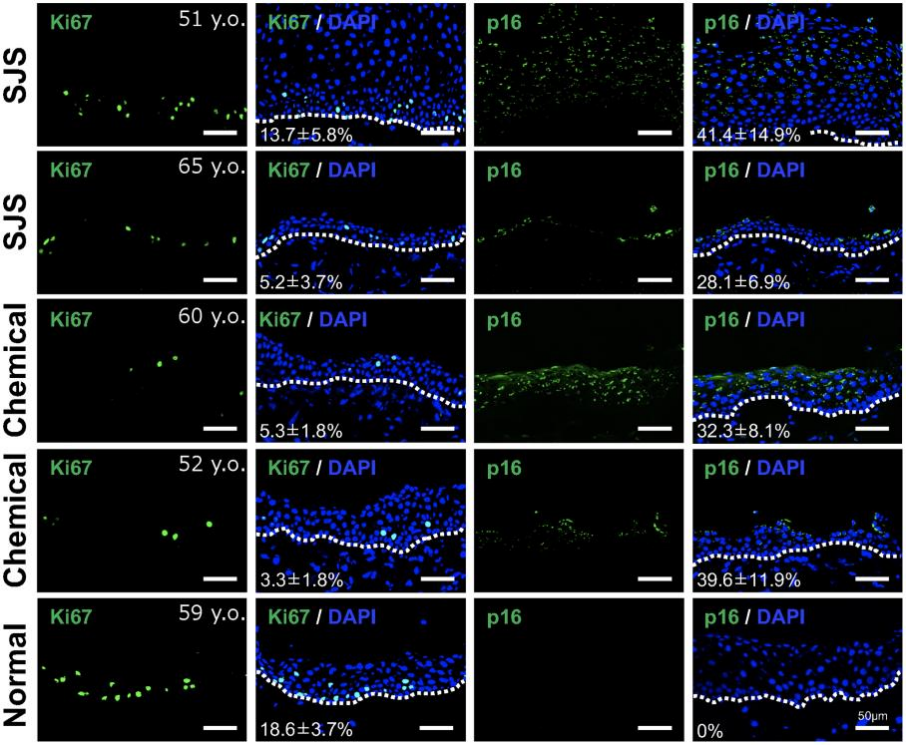
**Presence of senescent cells in pathological conjunctival epithelium.** Immunostaining of p16 and Ki67 in patients with Stevens-Johnson syndrome (SJS) or chemical injury, and in healthy individual. The positivity rate was calculated by quantifying the percentage of positive cells per sample (mean ± standard deviation) based on five independent fields. Scale bars indicate 50 μm.

We next investigated whether senescent conjunctival epithelial cells could be involved in the pathogenesis of LSCD caused by Stevens-Johnson syndrome or chemical burns. Limbal stem cells are located at the border between cornea and conjunctiva, and continuously produce and replenish corneal epithelial cells toward the center of the cornea, thereby maintaining the homeostasis of the corneal epithelium. In the case of LSCD, corneal epithelial stem cells are depleted, allowing conjunctival epithelium to invade and induce pathological vascularization, leading to significant corneal opacification (Figure 4). We collected pathological conjunctival tissues on the ocular surface at the time of surgery, and compared them with normal conjunctival tissues from individuals at the same age. p16-positivity in the conjunctival epithelium was not observed in normal conjunctiva, but appeared in the superficial epithelial cells of the pathological conjunctiva (Figure 5). On the other hand, ki67-positive cells were observed in the basal layer in both cases, but the number was reduced in LSCD compared to normal conjunctiva (Figure 5).

**Figure 6:**
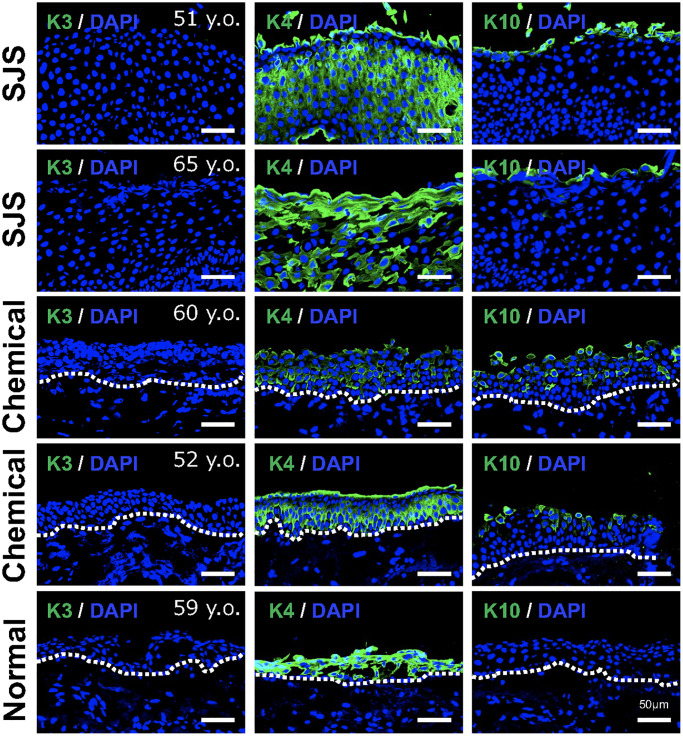
**Expression pattern of keratins in pathological conjunctival epithelium.** Immunostaining of keratin 3 (K3), keratin 4 (K4), and keratin 10 (K10) in patients with Stevens-Johnson syndrome (SJS) or chemical injury, and in healthy individual. Scale bars indicate 50 μm.

As shown in Figure 2D, gene expression related to epithelial differentiation, such as keratinocyte differentiation and keratinization, was enriched in SCj. Therefore, we next examined the expression pattern of several keratins. We found that keratin 3 was not expressed in any patient tissue, whereas keratin 4 was strongly expressed in all conjunctival tissues including pathological tissues. Interestingly, pathological conjunctival tissues derived from patients expressing p16 showed expression of keratin 10, a keratinocyte-specific keratin, which was not observed in normal conjunctiva (Figure 6). These results suggest that senescent conjunctival epithelial cells are involved in abnormal epithelial differentiation and contribute to the pathogenesis of conjunctival tissues.

## DISCUSSION

We performed a comprehensive analysis of RNA expression in human corneal and conjunctival epithelial cells induced to senescence upon X-irradiation. We found that both SCo and SCj not only showed high expression of typical markers of cellular senescence, but also exhibited a SASP, which included inflammatory cytokines and chemokines previously observed in other cell types [5]. Moreover, compared to SCo, SCj displayed a stronger increase in expression of genes associated with keratinocyte differentiation and keratinization. These results suggest the potential existence of distinct characteristics of cellular senescence between the cornea and conjunctiva.

Previous research on senescence in the ocular surface reported in the elderly that p16-positive senescent cells were present in the superficial layer of the conjunctival epithelium, while the number of proliferative cells in the basal layer was reduced. Additionally, the conjunctival epithelium of elderly individuals, which exhibited increased p16 expression, showed a loss in ZO-1 expression [12]. Analysis of aged donor corneas also revealed the presence of p16 in the superficial layer of the corneal epithelium and an upregulation of TGF-β1 signaling activity [13]. The curated dataset of GO biological process annotations in our study included various processes involving the TGF-β2 gene, such as the regulation of cell proliferation, apoptotic process, cell migration, and cell differentiation. These data suggest that cellular senescence may contribute to abnormal differentiation at the ocular surface.

One of the most intriguing findings of our study was the distinct gene expression profile observed in SCj compared to SCo, despite the significant similarity between nSCo and nSCj. SCj display more pronounced abnormalities in cellular differentiation. In a previous report, we showed that p16 expression was almost completely absent in individuals in their 50 s, but emerged after the age of 70 [12]. However, upon examination of pathological conjunctival tissues, we detected a significant number of p16-positive cells, even in patients in their 50 s or 60 s. Additionally, alteration in keratin expression patterns was observed, suggesting the involvement of senescent cells in the differentiation abnormalities of pathological tissues. These findings may indicate that ocular surface cells exposed to acute destructive inflammation in patients with LSCD, caused by Stevens-Johnson syndrome or chemical burns, are subject to high levels of stress, thereby accelerating the aging process of the ocular surface.

It was previously documented that cellular senescence is not only triggered by X-irradiation, oncogenes and chemical substances, but also by ultraviolet (UV) irradiation [14]. However, it is important to note that UVA and UVB have distinct effects on cellular senescence. UVA primarily induces secondary DNA damage through oxidative stress, resulting in the induction of cellular senescence, whereas UVB directly interacts with DNA and generates dipyrimidine photoproducts, causing cellular senescence. Moreover, senescent cells can exhibit significantly different profiles of SASP depending on the specific inducers of cellular senescence, even within the same cell type [15]. Therefore, further investigations are required to investigate cellular senescence at the ocular surface, induced by other factors such as UVA and UVB.

Overall, our results from the RNA-Seq experiments show that senescent ocular surface cells, particularly SCj, have abnormal keratin expression patterns, highlighting their involvement in the pathological features happening at the ocular surface.

## MATERIALS AND METHODS

### Cell cultures

Human primary CoEpiCs and CjEpiCs were obtained from donor corneas (CorneaGen, Seattle Eye Bank, Seattle, WA, USA). The cells were isolated and cultured following a previously described method [16] with slight modifications. The corneal and conjunctival epithelium were peeled off after overnight treatment with dispase type II (Godo Shusei, Tokyo, Japan) at a concentration of 1000 PU/mL at 4°C. The epithelial cells were then dissociated into single cells by incubation with TrypLE™ Express (Thermo Fisher Scientific, Waltham, MA, USA) for 5 min at 37°C and seeded into a single well of a 6-well plate. The CoEpiCs and CjEpiCs were cultured at 37°C, 95% humidity, and 5% CO_2_ in complete medium [17], consisting of Dulbecco’s modified Eagle’s medium and Ham’s F-12 media (DMEM/F12, 1:1 mixture) (Thermo Fisher Scientific), B-27™ supplement (2%) (Thermo Fisher Scientific), Rho-kinase inhibitor Y-27632 (10 μM) (Selleck Chemicals, Houston, TX, USA), keratinocyte growth factor (10 ng/ml) (Thermo Fisher Scientific), and penicillin-streptomycin (50 IU/ml) (Nacalai Tesque, Kyoto, Japan). The cells were used for subsequent experiments after one passage.

### Senescence induction

Senescent CoEpiCs and CjEpiCs were induced using ionizing radiation (IR; 10 Gy X-ray) according to previous reports [10, 18]. The irradiated cells were cultured for 10 days to allow the development of senescent characteristics. Mock radiation was applied to cells as a control treatment.

### SA-β-gal staining and EdU labeling

The cells were fixed using a fixation solution and subjected to senescence-associated β-galactosidase (SA-β-gal) staining following the manufacturer’s instructions (Biovision, Waltham, MA, USA). Imaging was performed using a Nikon Eclipse E800 microscope, and the number of positive cells was recorded. Cell proliferation was assessed by incorporating 5-ethynyl-2′-deoxyuridine (EdU) using the Click-iT EdU Cell Proliferation Assay Kit (Invitrogen). Quantification was performed using FIJI/ImageJ software (Version: 2.9.0/1.53t).

### qRT-PCR analysis

Total RNA was extracted using the Bioline Isolate II RNAMini Kit (Meridian Bioscience, Cincinnati, OH, USA) following the manufacturer’s protocol. Complementary DNA (cDNA) was synthesized from 500 ng of total RNA using the High-Capacity cDNA Reverse Transcription Kit (Thermo Fisher Scientific). The primer sets for the genes of interest are provided in Table 1. A reaction mixture with cDNA, primer and THUNDERBIRD^®^ SYBR^®^ qPCR Mix (TOYOBO Corp., Osaka, Japan) was amplified using a QuantStudio™ 3 instrument (Applied Biosystems, Waltham, MA). The relative expression levels of each gene were normalized to the housekeeping gene GAPDH and calculated by comparing the Ct values of the target genes with GAPDH.

**Table 1:** List of oligomers used for this study.

**Primer**	**Sequence**
P21-forward	5′-TGTCCGTCAGAACCCATGC-3′
P21-reverse	5′-AAAGTCGAAGTTCCATCGCTC-3′
P16-forward	5′-ATGGAGCCTTCGGCTGACT-3′
P16-reverse	5′-GTAACTATTCGGTGCGTTGGG-3′
GAPDH-forward	5′-GAAGGTGAAGGTCGGAGT-3′
GAPDH-reverse	5′-GAAGATGGTGATGGGATTTC-3′

### Western blotting

Cells were rinsed with ice-cold PBS and then lysed in RIPA buffer supplemented with 6% of 2-mercaptoethanol and Halt Protease and Phosphatase Inhibitor Cocktail (Thermo Fisher Scientific). The lysates were subsequently boiled at 95°C for 5 min. Equal amounts (20 μg) of the lysate samples were loaded onto 4–12% Bis-Tris gels (Bio-Rad, Hercules, CA, USA) for SDS-PAGE separation. The proteins were then transferred to PVDF membranes using the iBlot Dry Blotting Gel Transfer System (Thermo Fisher Scientific). Following transfer, the membranes were blocked for 1 h at room temperature in 5% BSA-TBST and then incubated with primary antibodies overnight at 4°C. After washing, the membranes were incubated with HRP-conjugated secondary antibodies for 1 h at room temperature and visualized using enhanced chemiluminescence. The primary antibodies were p16 (Abcam, #ab108349, anti-rabbit, 1:500 dilution). The secondary antibodies were HRP-conjugated goat anti-rabbit and goat anti-mouse (Bio-Rad). Beta-actin (Sigma, #A2228, anti-mouse, 1:10,000 dilution) was used as an internal control.

### RNA-Seq analysis

We extracted RNAs from SCo and SCj as well as nSCo and nSCj (3 replicates per condition) obtained from a 74-year old donor to perform RNA-Seq analysis. To evaluate the quality of the raw RNA-seq data, we initially performed a quality assessment using FastQC (v0.12.1; https://www.bioinformatics.babraham.ac.uk/projects/fastqc/). To remove adapter sequences and low-quality reads, we used TrimGalore (v4.3; https://www.bioinformatics.babraham.ac.uk/projects/trim_galore/) [19]. The resulting trimmed reads were aligned to the reference genome (GRCh38) using HISAT2 (v2.2.1) [20]. Subsequently, we converted the output SAM files to sorted BAM files using SAMtools (v1.12) [21] for downstream analysis. Mapped reads were assigned to their corresponding genes using FeatureCounts (v2.0.3) [22], which generated count data for each gene. The raw read counts were then normalized using DESeq2 (v1.8.3) [23]. For exploratory analysis, differential gene expression analysis, Kyoto Encyclopedia of Genes and Genomes (KEGG) pathway analysis, and enrichment analysis, we utilized iDEP [24]. Differential gene expression analysis was performed using DESeq2 (v1.8.3) in R (v4.2.3; https://www.R-project.org/) and RStudio (v2023.03.0 + 386; https://www.rstudio.com/). Gene ontology (GO) and pathway analysis for the identified differentially expressed genes (DEGs) were conducted using the clusterProfiler (v4.6.2) package [25].

### Human sample collection and hematoxylin and eosin (HE) staining

Conjunctival tissue samples were collected from two patients with Stevens-Johnson syndrome, two patients with chemical burns at the time of surgery, and one normal subject as a control. The surgeries took place at the University of Hospital, Kyoto Prefectural University of Medicine in Kyoto, Japan. The collected samples were immediately embedded in Tissue-Tek^®^ O.C.T. Compound (Sakura Finetek Japan Co., Ltd, Tokyo, Japan) and cryopreserved at −80°C. The collection of samples followed the ethical guidelines outlined in the Declaration of Helsinki and was approved by the Research Ethics Committee of Kyoto Prefectural University of Medicine (#ERB-C-1522). The tissue samples were fixed in 4% paraformaldehyde, stained with Meyer’s hematoxylin and eosin solution, dehydrated, and immersed in xylene as previously reported [26], and images were captured using a microscope (DP80 TRF; Olympus Corp., Tokyo, Japan).

### Immunofluorescence staining

Cryopreserved human conjunctival tissues were sectioned at a thickness of 7 μm and fixed using Zamboni’s fixative (a phosphate-buffered combination of picric acid and paraformaldehyde) at a temperature of 4°C for a duration of 10 min. Subsequently, the samples were washed and permeabilized with 0.3% Triton-X-100 at room temperature for 15 min. The samples were then blocked using Blocking One (Nacalai Tesque, Kyoto, Japan) and incubated overnight with the primary antibody at a temperature of 4°C. On the following day, the samples were washed with PBS and incubated at room temperature for 1 h with the secondary antibody (Alexa Fluor 488-labeled anti-mouse IgG, Life Technologies Corp., Carlsbad, CA, USA) at a dilution of 1:1000. Finally, after washing, DAPI was added, and the signal was visualized using a confocal laser microscope (FV1200-SIR-W5, Olympus Corp., Tokyo, Japan). The primary antibodies used were p16 (Enzo, #ENZ-ABS377, anti-mouse, 1:100 dilution), ki67 (Dako, #M7240, anti-mouse, 1:100 dilution), K3 (PROGEN, #61807, anti-mouse, 1:100 dilution), K4 (SantaCruz, #sc-52321, anti-mouse, 1:100 dilution), and K10 (Abcam, #ab9025, anti-mouse, 1:100 dilution). The percentage of positive cells per sample (mean ± standard deviation) was calculated based on five independent fields.

### Statistics

Mean ± S.E.M. values are shown for all data, and individual data points are represented in the bar graphs. Statistical analyses were performed using Prism 9 software version 9.4.1 (458) (GraphPad, La Jolla, CA, USA). Welch’s adjusted *t*-test (also known as unequal variances *t*-test), a modified version of Student’s *t*-test, was also used assuming unequal variances. Cell culture experiments were mostly performed in triplicate and independently replicated at least three times.

### Data availability statement

Raw data set for RNA-Seq was deposited in GEO (accession number: GSE232508 and GSE237091).

## Supplementary Materials

Supplementary Figure 1
