# Supplementary figures and images for: Gene expression signatures of human senescent corneal and conjunctival epithelial cells
Source: Aging (Albany NY). 2023 Sep 28;15(18):9238–49. doi: 10.18632/aging.205113 (PMC10564427; doi:10.18632/aging.205113)

[www.aging-us.com](http://www.aging-us.com)

1

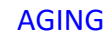

www.aging-us.com 1 AGING

Supplement: Supplementary Figure 1 [file aging-15-205113-s001.pdf]
